# Supplementary material for: Modeling glioblastoma heterogeneity as a dynamic network of cell states
Source: Mol Syst Biol. 2021 Sep 16;17(9):e10105. doi: 10.15252/msb.202010105 (PMC8444284; doi:10.15252/msb.202010105)
Supplement: Supplementary file 6 — Source Data for Figure 5 [file MSB-17-e10105-s004.zip › Figure5A_sourcedata/GSEA_3071/hallmarks_stateI.GseaPreranked.1623742531165/HALLMARK_GLYCOLYSIS.html]

Details for gene set HALLMARK\_GLYCOLYSIS[GSEA]

|  || Dataset | state13071 |
| Phenotype | NoPhenotypeAvailable |
| Upregulated in class | na\_pos |
| GeneSet | HALLMARK\_GLYCOLYSIS |
| Enrichment Score (ES) | 0.33499306 |
| Normalized Enrichment Score (NES) | 1.8147411 |
| Nominal p-value | 0.0063091484 |
| FDR q-value | 0.011570953 |
| FWER p-Value | 0.091 |
Table: GSEA Results Summary

  

Fig 1: Enrichment plot: HALLMARK\_GLYCOLYSIS      
 Profile of the Running ES Score & Positions of GeneSet Members on the Rank Ordered List

  

| PROBE | GENE SYMBOL | GENE\_TITLE | RANK IN GENE LIST | RANK METRIC SCORE | RUNNING ES | CORE ENRICHMENT || 1 | DCN |  |  | 5 | 2.010 | 0.0459 | Yes |
| 2 | COL5A1 |  |  | 14 | 1.706 | 0.0838 | Yes |
| 3 | TGFBI |  |  | 17 | 1.674 | 0.1227 | Yes |
| 4 | TFF3 |  |  | 18 | 1.667 | 0.1620 | Yes |
| 5 | TPBG |  |  | 51 | 1.346 | 0.1844 | Yes |
| 6 | P4HA2 |  |  | 82 | 1.171 | 0.2033 | Yes |
| 7 | NDST3 |  |  | 103 | 1.082 | 0.2230 | Yes |
| 8 | KDELR3 |  |  | 136 | 0.951 | 0.2361 | Yes |
| 9 | EGFR |  |  | 175 | 0.864 | 0.2454 | Yes |
| 10 | PYGB |  |  | 187 | 0.849 | 0.2622 | Yes |
| 11 | VCAN |  |  | 197 | 0.833 | 0.2793 | Yes |
| 12 | PLOD1 |  |  | 271 | 0.713 | 0.2749 | Yes |
| 13 | P4HA1 |  |  | 291 | 0.688 | 0.2856 | Yes |
| 14 | UGP2 |  |  | 318 | 0.657 | 0.2935 | Yes |
| 15 | HSPA5 |  |  | 337 | 0.638 | 0.3033 | Yes |
| 16 | GFPT1 |  |  | 403 | 0.585 | 0.2982 | Yes |
| 17 | TALDO1 |  |  | 455 | 0.545 | 0.2963 | Yes |
| 18 | CHPF2 |  |  | 460 | 0.543 | 0.3079 | Yes |
| 19 | SDC3 |  |  | 471 | 0.534 | 0.3176 | Yes |
| 20 | GLRX |  |  | 485 | 0.524 | 0.3262 | Yes |
| 21 | ME1 |  |  | 543 | 0.484 | 0.3210 | Yes |
| 22 | SLC16A3 |  |  | 570 | 0.470 | 0.3246 | Yes |
| 23 | SRD5A3 |  |  | 573 | 0.467 | 0.3350 | Yes |
| 24 | G6PD |  |  | 671 | 0.423 | 0.3168 | No |
| 25 | AKR1A1 |  |  | 679 | 0.421 | 0.3247 | No |
| 26 | HDLBP |  |  | 696 | 0.413 | 0.3298 | No |
| 27 | DDIT4 |  |  | 865 | 0.361 | 0.2895 | No |
| 28 | RBCK1 |  |  | 879 | 0.356 | 0.2941 | No |
| 29 | GPC1 |  |  | 882 | 0.354 | 0.3019 | No |
| 30 | CACNA1H |  |  | 964 | 0.335 | 0.2863 | No |
| 31 | MET |  |  | 974 | 0.333 | 0.2915 | No |
| 32 | EGLN3 |  |  | 988 | 0.329 | 0.2955 | No |
| 33 | GCLC |  |  | 1064 | 0.314 | 0.2811 | No |
| 34 | PLOD2 |  |  | 1304 | 0.268 | 0.2180 | No |
| 35 | CD44 |  |  | 1430 | -0.251 | 0.1877 | No |
| 36 | GALE |  |  | 1592 | -0.267 | 0.1472 | No |
| 37 | ALDH7A1 |  |  | 1615 | -0.269 | 0.1472 | No |
| 38 | NDUFV3 |  |  | 1618 | -0.270 | 0.1529 | No |
| 39 | VEGFA |  |  | 1644 | -0.273 | 0.1521 | No |
| 40 | ENO2 |  |  | 1707 | -0.281 | 0.1407 | No |
| 41 | CITED2 |  |  | 1782 | -0.291 | 0.1261 | No |
| 42 | TPI1 |  |  | 1828 | -0.297 | 0.1200 | No |
| 43 | ARPP19 |  |  | 1837 | -0.298 | 0.1247 | No |
| 44 | PAM |  |  | 1959 | -0.313 | 0.0970 | No |
| 45 | ME2 |  |  | 2053 | -0.326 | 0.0777 | No |
| 46 | PYGL |  |  | 2071 | -0.329 | 0.0805 | No |
| 47 | CENPA |  |  | 2220 | -0.357 | 0.0460 | No |
| 48 | PRPS1 |  |  | 2267 | -0.366 | 0.0412 | No |
| 49 | HOMER1 |  |  | 2313 | -0.374 | 0.0370 | No |
| 50 | PPP2CB |  |  | 2384 | -0.390 | 0.0258 | No |
| 51 | AURKA |  |  | 2467 | -0.404 | 0.0116 | No |
| 52 | EXT1 |  |  | 2615 | -0.443 | -0.0207 | No |
| 53 | GPC4 |  |  | 2653 | -0.455 | -0.0207 | No |
| 54 | AK3 |  |  | 2716 | -0.475 | -0.0275 | No |
| 55 | IRS2 |  |  | 2821 | -0.509 | -0.0457 | No |
| 56 | FKBP4 |  |  | 2871 | -0.528 | -0.0475 | No |
| 57 | POLR3K |  |  | 2872 | -0.528 | -0.0350 | No |
| 58 | CAPN5 |  |  | 2880 | -0.531 | -0.0245 | No |
| 59 | SAP30 |  |  | 3096 | -0.671 | -0.0711 | No |
| 60 | NASP |  |  | 3106 | -0.682 | -0.0576 | No |
| 61 | CDK1 |  |  | 3149 | -0.727 | -0.0527 | No |
| 62 | STMN1 |  |  | 3171 | -0.751 | -0.0411 | No |
| 63 | B3GAT1 |  |  | 3175 | -0.754 | -0.0242 | No |
| 64 | CXCR4 |  |  | 3217 | -0.803 | -0.0172 | No |
| 65 | SOX9 |  |  | 3271 | -0.883 | -0.0117 | No |
| 66 | CTH |  |  | 3336 | -0.990 | -0.0070 | No |
| 67 | STC1 |  |  | 3349 | -1.043 | 0.0141 | No |
| 68 | IGFBP3 |  |  | 3433 | -1.384 | 0.0226 | No |
Table: GSEA details [plain text format]

  

Fig 2: HALLMARK\_GLYCOLYSIS: Random ES distribution      
 Gene set null distribution of ES for **HALLMARK\_GLYCOLYSIS**
